# Supplementary material for: Deletion of wfs1 Impairs Oligodendrocyte Precursor Cells Dorsal Distribution and Myelination Through the wfs1-hmgcs1 Axis in Zebrafish
Source: Biology (Basel). 2026 Aug 16;15(16):1402. doi: 10.3390/biology15161402 (PMC13510151; doi:10.3390/biology15161402)
Supplement: Supplementary file 1 [file biology-15-01402-s001.zip › File S1-Transcriptome sequencing data and differentially expressed genes (DEGs) in wfs1-- zebrafish at 5 dpf/2.KEGG/wfs1KOvsWT/GSEA/WT/MITOPHAGY___ANIMAL/MITOPHAGY___ANIMAL(DRE04137).html]

Details for gene set MITOPHAGY\_\_\_ANIMAL(DRE04137)[GSEA]

|  || Dataset | deg.sample |
| Phenotype | sample.cls |
| Upregulated in class | WT |
| GeneSet | MITOPHAGY\_\_\_ANIMAL(DRE04137) |
| Enrichment Score (ES) | -0.26127002 |
| Normalized Enrichment Score (NES) | -1.2256162 |
| Nominal p-value | 0.13195877 |
| FDR q-value | 0.49595398 |
| FWER p-Value | 1.0 |
Table: GSEA Results Summary

  

Fig 1: Enrichment plot: MITOPHAGY\_\_\_ANIMAL(DRE04137)      
 Profile of the Running ES Score & Positions of GeneSet Members on the Rank Ordered List

  

| PROBE | DESCRIPTION (from dataset) | GENE SYMBOL | GENE\_TITLE | RANK IN GENE LIST | RANK METRIC SCORE | RUNNING ES | CORE ENRICHMENT || 1 | 550134\_1 | ubb |  |  | 694 | 2.603 | 0.0117 | No |
| 2 | 556410\_1 | atf4b |  |  | 780 | 2.425 | 0.0403 | No |
| 3 | novel.905 | - |  |  | 864 | 2.303 | 0.0674 | No |
| 4 | 555713 | nbr1b |  |  | 866 | 2.301 | 0.0971 | No |
| 5 | 564996 | tfe3b |  |  | 963 | 2.143 | 0.1217 | No |
| 6 | 492676 | tbc1d15 |  |  | 1206 | 1.890 | 0.1385 | No |
| 7 | 326974\_1 | gabarapa |  |  | 1391 | 1.732 | 0.1550 | No |
| 8 | 561933\_2 | rhot1b |  |  | 1819 | 1.452 | 0.1603 | No |
| 9 | 777766 | ubc |  |  | 1935 | 1.395 | 0.1747 | No |
| 10 | 559564\_1 | eif2ak3 |  |  | 2076 | 1.336 | 0.1875 | No |
| 11 | 561933\_1 | rhot1b |  |  | 2587 | 1.149 | 0.1863 | No |
| 12 | 559355 | bcl2l13 |  |  | 2713 | 1.106 | 0.1966 | No |
| 13 | 30590\_1 | tp53 |  |  | 2944 | 1.051 | 0.2029 | No |
| 14 | 100000242\_1 | zgc:92606 |  |  | 3071 | 1.020 | 0.2121 | No |
| 15 | 494085 | pink1 |  |  | 3376 | 0.958 | 0.2149 | No |
| 16 | 562289 | tfeb |  |  | 4184 | 0.796 | 0.1998 | No |
| 17 | 336116 | bnip3 |  |  | 4475 | 0.748 | 0.2003 | No |
| 18 | 101885420 | e2f1 |  |  | 4903 | 0.693 | 0.1958 | No |
| 19 | 567448 | mfn2 |  |  | 5011 | 0.680 | 0.2012 | No |
| 20 | 100330105 | map1lc3cl |  |  | 5232 | 0.652 | 0.2027 | No |
| 21 | 114834 | tfe3a |  |  | 5609 | 0.625 | 0.1989 | No |
| 22 | 554650 | bnip3lb |  |  | 6539 | 0.533 | 0.1766 | No |
| 23 | 325084 | src |  |  | 6875 | 0.502 | 0.1725 | No |
| 24 | 65236 | mapk8b |  |  | 6998 | 0.492 | 0.1750 | No |
| 25 | 335916 | jun |  |  | 7293 | 0.467 | 0.1718 | No |
| 26 | 110438533 | LOC110438533 |  |  | 7645 | 0.443 | 0.1664 | No |
| 27 | 393902\_2 | rab7 |  |  | 8135 | 0.416 | 0.1564 | No |
| 28 | 791223 | atg9b |  |  | 8583 | 0.396 | 0.1475 | No |
| 29 | 751649 | tomm7 |  |  | 8982 | 0.369 | 0.1397 | No |
| 30 | 336159 | optn |  |  | 9027 | 0.366 | 0.1430 | No |
| 31 | 337810 | rps27a |  |  | 9031 | 0.365 | 0.1477 | No |
| 32 | 559099 | usp30 |  |  | 9150 | 0.357 | 0.1485 | No |
| 33 | 402792 | zgc:73226 |  |  | 9206 | 0.353 | 0.1514 | No |
| 34 | 415254 | zgc:86598 |  |  | 9372 | 0.343 | 0.1506 | No |
| 35 | 797988 | fis1 |  |  | 9600 | 0.327 | 0.1477 | No |
| 36 | 406466 | map1lc3a |  |  | 9731 | 0.319 | 0.1477 | No |
| 37 | 562720 | mapk8a |  |  | 9888 | 0.310 | 0.1468 | No |
| 38 | 793200 | gabarapb |  |  | 9960 | 0.306 | 0.1485 | No |
| 39 | 337810\_1 | rps27a |  |  | 10695 | 0.261 | 0.1288 | No |
| 40 | 393228\_1 | zgc:55558 |  |  | 10754 | 0.257 | 0.1302 | No |
| 41 | 323563 | bnip3la |  |  | 10852 | 0.252 | 0.1304 | No |
| 42 | 550513\_1 | rras2 |  |  | 10897 | 0.250 | 0.1323 | No |
| 43 | 559106 | ambra1b |  |  | 11593 | 0.211 | 0.1131 | No |
| 44 | 556410\_2 | atf4b |  |  | 12071 | 0.187 | 0.1005 | No |
| 45 | 431725 | zgc:91909 |  |  | 12321 | 0.172 | 0.0949 | No |
| 46 | 100332642 | ambra1a |  |  | 12365 | 0.171 | 0.0958 | No |
| 47 | 494576\_1 | si:dkey-13a21.4 |  |  | 12522 | 0.163 | 0.0930 | No |
| 48 | 561219 | tax1bp1a |  |  | 12762 | 0.151 | 0.0874 | No |
| 49 | 103909433 | LOC103909433 |  |  | 13029 | 0.138 | 0.0808 | No |
| 50 | 393902\_1 | rab7 |  |  | 13530 | 0.114 | 0.0665 | No |
| 51 | 100170793 | ulk1a |  |  | 13675 | 0.108 | 0.0634 | No |
| 52 | 114401 | bcl2l1 |  |  | 13894 | 0.097 | 0.0578 | No |
| 53 | 569698 | mapk10 |  |  | 13907 | 0.096 | 0.0587 | No |
| 54 | 556379 | csnk2a2b |  |  | 14295 | 0.078 | 0.0475 | No |
| 55 | 393202 | hif1ab |  |  | 14484 | 0.070 | 0.0425 | No |
| 56 | 100009663 | atg9a |  |  | 14551 | 0.067 | 0.0413 | No |
| 57 | 494180 | atg5 |  |  | 14638 | 0.063 | 0.0394 | No |
| 58 | 100536317\_1 | si:ch73-116o1.2 |  |  | 14750 | 0.058 | 0.0366 | No |
| 59 | 554092 | tbc1d17 |  |  | 14929 | 0.050 | 0.0317 | No |
| 60 | 568155\_1 | bnip4 |  |  | 15432 | 0.025 | 0.0162 | No |
| 61 | 30296 | foxo3b |  |  | 15498 | 0.022 | 0.0144 | No |
| 62 | 403036 | gabarapl2 |  |  | 15513 | 0.022 | 0.0143 | No |
| 63 | 30380 | nras |  |  | 15635 | 0.015 | 0.0107 | No |
| 64 | 558848 | ulk1b |  |  | 17219 | -0.042 | -0.0386 | No |
| 65 | 337168 | nbr1a |  |  | 17247 | -0.043 | -0.0389 | No |
| 66 | 565434 | usp8 |  |  | 17506 | -0.054 | -0.0463 | No |
| 67 | 100149273 | mapk9 |  |  | 17865 | -0.070 | -0.0567 | No |
| 68 | 449660 | rras |  |  | 18718 | -0.109 | -0.0821 | No |
| 69 | 553776 | calcoco2 |  |  | 18782 | -0.112 | -0.0826 | No |
| 70 | 415099 | rela |  |  | 18848 | -0.116 | -0.0832 | No |
| 71 | 557574 | rhot2 |  |  | 18918 | -0.118 | -0.0838 | No |
| 72 | 30488 | csnk2a2a |  |  | 19129 | -0.128 | -0.0888 | No |
| 73 | 492357 | pgam5 |  |  | 19705 | -0.153 | -0.1049 | No |
| 74 | 30502 | csnk2a1 |  |  | 19706 | -0.153 | -0.1030 | No |
| 75 | 30428 | csnk2b |  |  | 19758 | -0.155 | -0.1026 | No |
| 76 | 393620 | mfn1b |  |  | 19941 | -0.163 | -0.1062 | No |
| 77 | 641289 | uba52 |  |  | 20156 | -0.173 | -0.1107 | No |
| 78 | 450024 | cited2 |  |  | 20396 | -0.183 | -0.1158 | No |
| 79 | 494576 | si:dkey-13a21.4 |  |  | 20660 | -0.197 | -0.1216 | No |
| 80 | 550513 | rras2 |  |  | 20916 | -0.211 | -0.1269 | No |
| 81 | 100535532 | csnk2a4 |  |  | 20986 | -0.215 | -0.1263 | No |
| 82 | 494532 | foxo3a |  |  | 21163 | -0.224 | -0.1289 | No |
| 83 | 393376 | hif1al |  |  | 21454 | -0.238 | -0.1350 | No |
| 84 | 406452 | sqstm1 |  |  | 22703 | -0.308 | -0.1703 | No |
| 85 | 324152 | tax1bp1b |  |  | 22896 | -0.319 | -0.1722 | No |
| 86 | 393846 | becn1 |  |  | 23266 | -0.345 | -0.1794 | No |
| 87 | 30080 | mitfa |  |  | 23560 | -0.364 | -0.1839 | No |
| 88 | 797150 | hif1aa |  |  | 24932 | -0.443 | -0.2213 | No |
| 89 | 324109 | sp1 |  |  | 25090 | -0.453 | -0.2204 | No |
| 90 | 100317924 | mras |  |  | 25337 | -0.469 | -0.2221 | No |
| 91 | 692289 | tbk1 |  |  | 25794 | -0.510 | -0.2299 | No |
| 92 | 550286 | hrasa |  |  | 26472 | -0.575 | -0.2438 | No |
| 93 | 393268 | map1lc3c |  |  | 27020 | -0.631 | -0.2529 | Yes |
| 94 | 100536317 | si:ch73-116o1.2 |  |  | 27244 | -0.646 | -0.2515 | Yes |
| 95 | 553656 | hrasb |  |  | 27554 | -0.680 | -0.2525 | Yes |
| 96 | 114833 | mitfb |  |  | 27688 | -0.699 | -0.2477 | Yes |
| 97 | 564996\_1 | tfe3b |  |  | 27833 | -0.720 | -0.2429 | Yes |
| 98 | 326974 | gabarapa |  |  | 27990 | -0.740 | -0.2383 | Yes |
| 99 | 436984 | fundc1 |  |  | 28027 | -0.746 | -0.2298 | Yes |
| 100 | 322425 | map1lc3b |  |  | 28058 | -0.750 | -0.2211 | Yes |
| 101 | 100537262 | LOC100537262 |  |  | 28366 | -0.798 | -0.2204 | Yes |
| 102 | 327143 | rhot1a |  |  | 28374 | -0.800 | -0.2103 | Yes |
| 103 | 559355\_1 | bcl2l13 |  |  | 28747 | -0.868 | -0.2108 | Yes |
| 104 | 550328 | park2 |  |  | 28752 | -0.869 | -0.1998 | Yes |
| 105 | 406514 | atf4a |  |  | 28789 | -0.876 | -0.1896 | Yes |
| 106 | 445289 | kras |  |  | 29269 | -0.994 | -0.1919 | Yes |
| 107 | 449549 | zgc:100918 |  |  | 29484 | -1.058 | -0.1849 | Yes |
| 108 | 336159\_1 | optn |  |  | 29806 | -1.166 | -0.1800 | Yes |
| 109 | 561933 | rhot1b |  |  | 29909 | -1.206 | -0.1677 | Yes |
| 110 | 492676\_1 | tbc1d15 |  |  | 30263 | -1.374 | -0.1610 | Yes |
| 111 | 30590 | tp53 |  |  | 30325 | -1.401 | -0.1449 | Yes |
| 112 | 559564 | eif2ak3 |  |  | 30600 | -1.590 | -0.1330 | Yes |
| 113 | 568155 | bnip4 |  |  | 30688 | -1.659 | -0.1143 | Yes |
| 114 | 555713\_1 | nbr1b |  |  | 31380 | -2.600 | -0.1026 | Yes |
| 115 | 550134 | ubb |  |  | 31783 | -4.479 | -0.0574 | Yes |
| 116 | 100000242 | zgc:92606 |  |  | 31809 | -4.711 | 0.0025 | Yes |
Table: GSEA details [plain text format]

  

Fig 2: MITOPHAGY\_\_\_ANIMAL(DRE04137)      
 Blue-Pink O' Gram in the Space of the Analyzed GeneSet

  

Fig 3: MITOPHAGY\_\_\_ANIMAL(DRE04137): Random ES distribution      
 Gene set null distribution of ES for **MITOPHAGY\_\_\_ANIMAL(DRE04137)**
